# Advancing implementation science through measure development and evaluation: a study protocol

**DOI:** 10.1186/s13012-015-0287-0

**Published:** 2015-07-22

**Authors:** Cara C. Lewis, Bryan J. Weiner, Cameo Stanick, Sarah M. Fischer

**Affiliations:** Department of Psychological and Brain Sciences, Indiana University, 1101 E. 10th St., Bloomington, IN 47405 USA; Department of Psychiatry and Behavioral Sciences, School of Medicine, Harborview Medical Center, University of Washington, 325 9th Ave, Box 359911, Seattle, WA 98104 USA; 1102-C McGavran-Greenberg Hall, University of North Carolina at Chapel Hill, 135 Dauer Drive, Campus Box 7411, Chapel Hill, NC 27599-7411 USA; Department of Psychology, University of Montana, 32 Campus Dr., Skaggs Bldg. 202, Missoula, MT 59812 USA

**Keywords:** Implementation science, Measures, Instruments, Psychometric, Pragmatic, Implementation outcomes, Consolidated Framework for Implementation Research

## Abstract

**Background:**

Significant gaps related to measurement issues are among the most critical barriers to advancing implementation science. Three issues motivated the study aims: (a) the lack of stakeholder involvement in defining pragmatic measure qualities; (b) the dearth of measures, particularly for implementation outcomes; and (c) unknown psychometric and pragmatic strength of existing measures. Aim 1: Establish a stakeholder-driven operationalization of pragmatic measures and develop reliable, valid rating criteria for assessing the construct. Aim 2: Develop reliable, valid, and pragmatic measures of three critical implementation outcomes, *acceptability*, *appropriateness*, and *feasibility*. Aim 3: Identify Consolidated Framework for Implementation Research and Implementation Outcome Framework-linked measures that demonstrate both psychometric and pragmatic strength.

**Methods/design:**

For Aim 1, we will conduct (a) interviews with stakeholder panelists (*N* = 7) and complete a literature review to populate pragmatic measure construct criteria, (b) Q-sort activities (*N* = 20) to clarify the internal structure of the definition, (c) Delphi activities (*N* = 20) to achieve consensus on the dimension priorities, (d) test-retest and inter-rater reliability assessments of the emergent rating system, and (e) known-groups validity testing of the top three prioritized pragmatic criteria. For Aim 2, our systematic development process involves domain delineation, item generation, substantive validity assessment, structural validity assessment, reliability assessment, and predictive validity assessment. We will also assess discriminant validity, known-groups validity, structural invariance, sensitivity to change, and other pragmatic features. For Aim 3, we will refine our established evidence-based assessment (EBA) criteria, extract the relevant data from the literature, rate each measure using the EBA criteria, and summarize the data.

**Discussion:**

The study outputs of each aim are expected to have a positive impact as they will establish and guide a comprehensive measurement-focused research agenda for implementation science and provide empirically supported measures, tools, and methods for accomplishing this work.

## Introduction

Poor-quality, impractical measurement has impeded the study of implementation barriers, facilitators, and strategies necessary for promoting widespread delivery of evidence-based care [1]. The identification of psychometrically strong and pragmatic measures has been identified as an international priority, requiring attention to three issues. First, while the need for pragmatic measures (that are both relevant to stakeholders and feasible for use in practice) has been emphasized and researchers have begun to describe the features that make measures pragmatic [2], it is unclear what *stakeholders* in practice (e.g., agency leaders, decision makers, purveyors, and clinicians) consider to be pragmatic measures or how they assign relative priority to various pragmatic qualities, such as brevity, simplicity of administration, and actionability. If stakeholder views are not taken into account, measurement developers run the risk of focusing on the wrong “pragmatic” attributes.

Second, few measures of implementation outcomes (e.g., feasibility, fidelity, and sustainability) have undergone systematic development and testing; as a result, the psychometric and pragmatic properties of most measures of implementation outcomes are not known. For example, of the 104 measures of implementation outcomes reviewed by the Society for Implementation Research Collaboration (SIRC), 51 % lacked information on reliability, 74 % on structural validity, 82 % on predictive validity, and 96 % on responsiveness. Implementation outcomes with many available measures (e.g., acceptability) had no more psychometrically and pragmatically strong measures as those implementation outcomes with few measures (e.g., feasibility) [3]. The dearth of reliable, valid, and pragmatic measures of implementation outcomes poses a significant barrier to advances in the field, since implementation outcomes are what we seek to explain in research and seek to achieve in practice.

Finally, it is unclear if measures can be both psychometrically strong and pragmatic as the majority of implementation related measures are of unknown psychometric and pragmatic qualities. Results from the four existing reviews [3–6] demonstrate that a range of 46 to 52 % of measures has not been psychometrically validated. And, only the Grid-Enabled Measures project includes a pragmatic measure evaluation; though, unfortunately their use of the crowd-sourcing methodology undermines the validity of their ratings [6]. Therefore, implementation researchers and practitioners have little guidance about which measures to select. Addressing these three issues is important because, if they are not addressed, implementation science runs the risk of “…constructing a magnificent house without bothering to build a solid foundation” [7] (i.e., reliable and valid measures) and replicating in measurement the science-practice gap that plagues intervention science [8] (i.e., pragmatic measures).

### Objectives and aims

The long-term objective of this research program is to make available to the field a comprehensive battery of reliable, valid, and pragmatic measures that researchers and stakeholders can use to advance implementation science and practice. To achieve this objective, three aims were articulated.

*Aim 1* Establish a stakeholder-driven operationalization of pragmatic measures and develop reliable, valid rating criteria for assessing the construct. Aim 1 will yield a stakeholder-driven operationalization of the pragmatic measures construct, an associated consensus-driven set of pragmatic rating criteria, and a reliable and valid method for evaluating measures for their pragmatic qualities.

*Aim 2* Develop reliable, valid, and pragmatic measures of three critical implementation outcomes, *acceptability*, *appropriateness*, and *feasibility*. Aim 2 will yield three high-quality, broadly applicable measures of implementation outcomes and a replicable, rigorous measure development process.

**Fig. 1:**
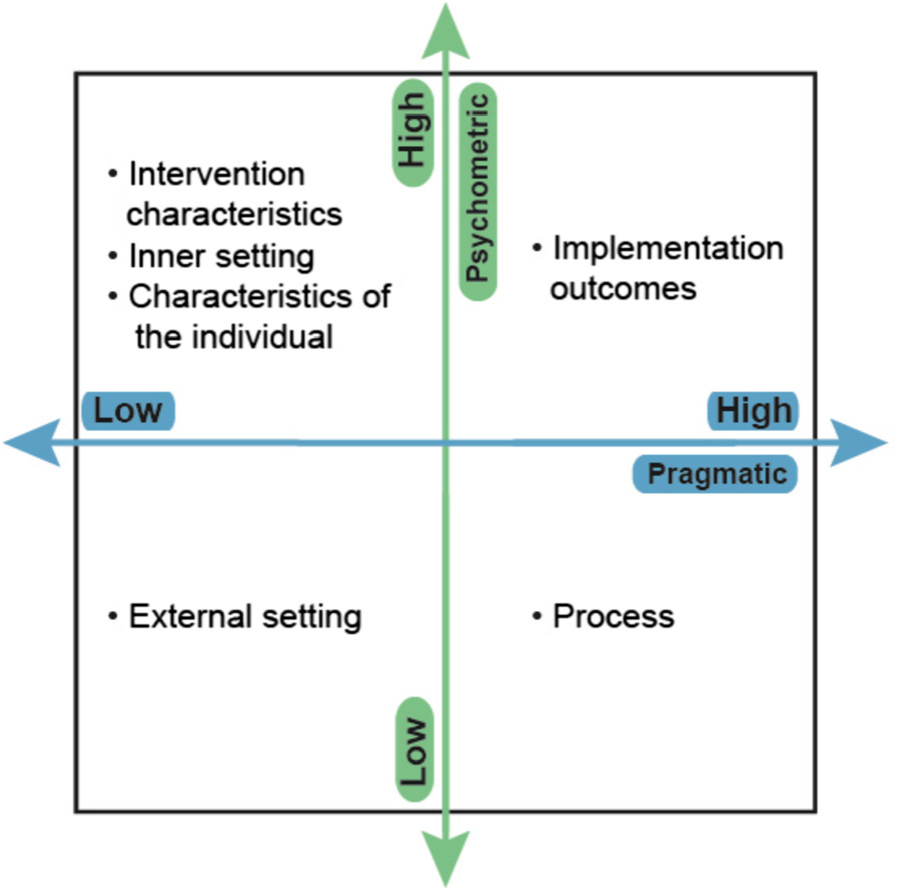
Psychometric-pragmatic grid. Note. Predictions of psychometric and pragmatic strength for measures across domains

*Aim 3* Identify Consolidated Framework for Implementation Research (CFIR) and Implementation Outcomes Framework (IOF)-linked measures that demonstrate both psychometric and pragmatic strength. We predict that measures of implementation outcomes, but no other constructs, will demonstrate psychometric and pragmatic strength (Fig. 1). Aim 3 will reveal which implementation domains (and constructs) possess high-quality measures and which require further development.

## Methods/design

### Incorporating key perspectives of leading implementation scientists and stakeholders

In 2006, it was noted that “implementation science” had 29 related terms in use around the globe [9], which threatens the cumulative knowledge that can be gained. To advance an implementation science measurement-focused research agenda, international perspectives must be incorporated. To ensure the broadest relevance of our work, we have created an international advisory board of nine implementation experts representing Australia (Sanson-Fisher and Wolfenden), Canada (Barwick and Grimshaw), the Netherlands (Wensing), Sweden (Ovretveit), the UK (Francis), and the USA (Damschroder and Mittman). The international advisory board will convene with the investigative team thrice annually to provide guidance on the project, contextualize findings, and contribute to the development of a measurement-focused research agenda. In addition, there is a 20-member task force, of PhD-level implementation scientists, that includes nationally recognized experts who will contribute to numerous studies within and across the three aims.

A recent observation suggests that a gap is growing between the findings from implementation science and the real-world practice of implementation [8], similar to the intervention science-practice gap which the field was developed to address. Early and consistent stakeholder involvement is critical to ensure relevance of research questions, methods, and measures. Thus, a stakeholder advisory panel of seven implementation champions representing state mental health, inpatient psychiatry, outpatient community mental health, school mental health, residential care, and low- and middle-income mental health settings (two of whom represent international perspectives), will advise and participate in Aim 1 studies and inform the work of Aims 2 and 3. In addition, there is a 20-member stakeholder task force who will contribute to numerous studies within and across the three aims.

### Aim 1: establish a stakeholder-driven operationalization of pragmatic measures and develop reliable, valid rating criteria for assessing the construct

#### Introduction

Practitioners are unlikely to use measures, no matter how psychometrically strong, if measures are not pragmatic—that is, relevant and feasible for use in real-world settings. Importantly, implementation science measures could aid stakeholders in prospectively assessing actionable barriers and facilitators, monitoring implementation impact, and feeding back implementation outcomes. As such, we will develop a stakeholder-driven operationalization and rating system to assess *pragmatic* measure qualities. Although stakeholder involvement in measure development is identified as an important *feature* of pragmatic measures [2], stakeholders have *not* driven the definition of the pragmatic construct itself. Further, no reliable and valid method exists for rating pragmatic measure qualities.

#### Research design

We will address this critical gap by assembling a stakeholder advisory panel (*N* = 7) and recruiting representative stakeholders (*N* = 20) to participate in a multi-tiered, mixed-methods data collection and refinement process. We will conduct (a) interviews with stakeholder panelists to populate pragmatic measure construct criteria through inductive qualitative inquiry, which will be combined with results from a literature review, (b) Q-sort activities (*N* = 20) to clarify the internal structure of the definition (grouping criteria into dimensions), (c) Delphi activities (*N* = 20) to achieve consensus on the dimension priorities (relative weights), (d) test-retest and inter-rater reliability assessments of the emergent rating system with multiple PhD-level implementation scientists, and (e) known-groups validity testing of the top three prioritized pragmatic criteria.

#### Domain delineation

We will conduct individual interviews with the stakeholder panel (two of whom represent international perspectives) to explore what pragmatic measurement means to them, what terms they use to describe pragmatic measures, and what attributes of measures they see are most and least relevant to the pragmatic construct. We will connect these inductively developed themes with deductively derived themes from a systematic literature review of the pragmatic construct (see Table 1). For instance, terms such as ‘usefulness’ or ‘perceived ease of use’ and ‘sensitivity to change’ have been identified in a preliminary literature review.

**Table 1:** Pragmatic dimensions

Required criteria	Definition
Important to stakeholders	Involve stakeholders from outset and on an ongoing basis
Burden is low for both respondents and staff	Usually brief and inexpensive
Actionable	Can correct identified problems or point to a specific intervention need
Sensitive to change	Can be used longitudinally to track progress and detect intervention effects

Pragmatic rating criteria; table contents reproduced from the work of Glasgow and Riley [2]

#### Clarifying the internal structure

We will use a modified Q-sort method (a bridge between qualitative and quantitative inquiry [10]) to clarify the internal structure of the pragmatic construct. Q-sort is an established method for detecting shared ways of thinking about partially overlapping and complex items [10]. Using survey software, the 20 stakeholder participants will sort items into the preliminary dimensions generated from the domain delineation. All participants will be asked to sort each of the items along a 10-point scale for those that are “most closely related to” each of the pragmatic dimensions to those “least closely related to” each dimension. Individual items that are ranked at 5 and above across all 20 stakeholders will be included in that dimension’s (e.g., low burden) defining characteristics (e.g., length, cost). The participants will also rate the degree to which each dimension is clear, precise, and distinct.

#### Achieving consensus on the criteria priorities

The final dimensions and associated items for the pragmatic rating criteria will be vetted through the 20 stakeholder participants utilizing a modified, multi-round, online Delphi method to achieve consensus on their relative weights [11]. The Delphi process engages a two-way iterative efficient exchange of information to achieve consensus [12]. In the first round, the dimensions (and associated items) from above will be submitted to the stakeholders who will distribute 100 points among the dimensions to reflect their relative importance for inclusion in the pragmatic rating criteria anchors. We will calculate the mean, median, mode, and interquartile range for each dimension and add this information to the web-based survey. In the first round, the stakeholders will again be asked to distribute 100 points among the dimensions, taking into account the measures of central tendency and levels of dispersion from the first round [13]. We will conduct Chi-square tests to determine whether stability in responses has been achieved; if not, a third (final) round will be engaged. The following characterization metric will be applied regarding stability of responses: *consensus* if unanimity is achieved, *majority* if 10 or more participants respond the same way, *plurality* if 5–9 stakeholders make up the largest subgroup, and *disagreement* if no subgroup response set has at least five members [14].

#### Developing the rating criteria

We will adapt dimensional standards from the evidence-based assessment (EBA) psychometric criteria developed for this proposal; its development is articulated in a previous publication [15]. That is, quantifiable pragmatic rating criteria will be developed wherein each dimension (and associated sub-dimensions) on which a measure may be rated will range from 0 (no evidence) to 4 (excellent). We will generate five-point anchors for each prioritized dimension of the pragmatic rating criteria.

#### Inter-rater, test-retest reliability, and known-groups assessment

The twenty PhD-level implementation scientist task force members will apply the pragmatic rating criteria to 12 measures, two from each of the six domains in the CFIR [16] and IOF. [17] To assess the criteria for known-groups validity, 6 of the 12 measures will be selected by the investigative team who will confirm obvious discrepancies. Five days later, the task force members will re-rate the measures. We will assess test-retest reliability using paired *t* tests and Pearson correlation coefficients and inter-rater reliability using intraclass correlations (ICCs). Scales demonstrating non-significant paired *t* test, correlations greater than 0.70 and ICCs greater than 0.60 will be considered reliable. A 3 × 2 within-subjects design will be used to assess the known-groups validity for the top three dimensions of the pragmatic criteria. Kappas will be calculated between the two raters; if kappas are ≥0.90 the dimension will be considered valid.

### Aim 2: develop reliable, valid, and pragmatic measures of acceptability, feasibility, and appropriateness

#### Introduction

We will develop reliable, valid, and pragmatic measures of the three implementation outcomes: acceptability, appropriateness, and feasibility. Proctor and her colleagues argue that acceptability, appropriateness, and feasibility are key implementation outcomes [17], which Wisdom indicates predict adoption [18]. Yet, none of the 66 measures of acceptability, appropriateness, or feasibility identified through SIRC or other reviews [4, 5] has undergone a systematic development process.

#### Research design

Our systematic development process follows the guidelines of measurement experts [19–21] and involves domain delineation, item generation, substantive validity assessment, structural validity assessment, reliability assessment, and predictive validity assessment. In addition, we will assess discriminant validity, known-groups validity, structural invariance, sensitivity to change, and other pragmatic features.

#### Domain delineation

**Fig. 2:**
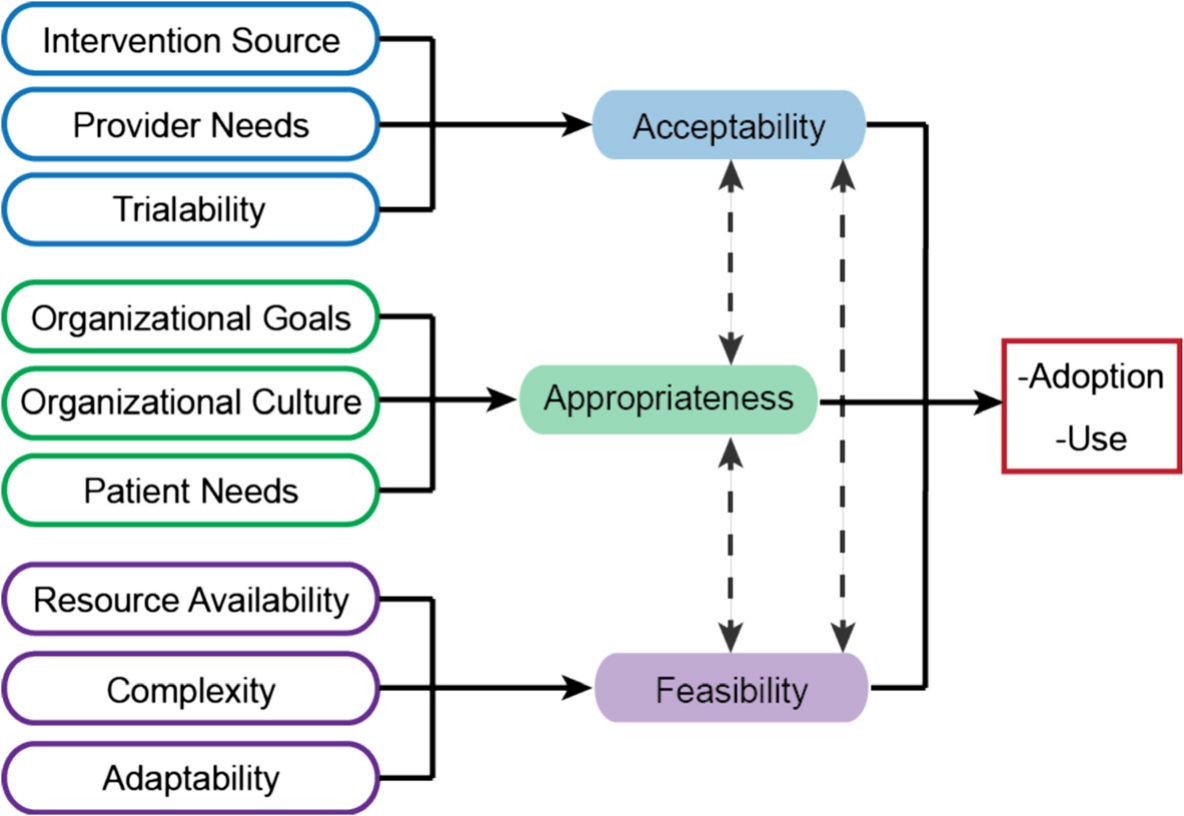
Nomological network. Note. Included in our nomological network are antecedent constructs that have high relevance for only one focal construct

Domain delineation enhances construct validity by reducing theoretical contamination in measures [19, 20]. Key steps involve developing conceptual definitions that distinguish the focal construct from related constructs and creating a nomological network (i.e., theoretically specifying relations among constructs). We offer the following *provisional* conceptual definitions and nomological network, which we will refine with input from our international advisory board. *Acceptability* refers to the extent to which an innovation or evidence-based practice (EBP) is attractive, agreeable, or palatable [17]. *Appropriateness* refers to the extent to which an innovation or EBP is suitable, fitting, or proper for a particular purpose or circumstance [17]. *Feasibility* refers to the extent to which an innovation or EBP is practical or possible to use. Generally, we view acceptability in terms of innovation-individual (provider) fit, appropriateness in terms of innovation-task fit and innovation-values (social) fit, and feasibility in terms of innovation-system fit. We include in our nomological network (Fig. 2) antecedent constructs that have high relevance for only one focal construct, rather than multiple constructs, in order to keep the network from becoming too complex and to guide the design of materials in the structural validity test.

#### Item generation

We will generate 10 items per construct assuming that half of the items will not survive psychometric testing and that 4–6 items per construct produce scales with sufficient internal consistency [21]. We will take a deductive approach to item generation [21], whereby we use the construct definitions and nomological network to ascertain whether items from existing measures adequately capture the theoretical content of the construct. We will write new items as needed to obtain 10 “direct measures” of each construct [22].

#### Substantive validity

Substantive validity is “the extent to which a measure is judged to be reflective of, or theoretically linked to, some construct of interest.” [23] When substantive validity is demonstrated for several constructs simultaneously, discriminant content validity is also demonstrated. Using procedures described by Huijg and colleagues [24], we *predict* two outcomes: (1) experts will assign 60 % or more of the generated items to their intended constructs, and (2) experts will exhibit substantial agreement in their assignments.

##### Design and sample

We will recruit 30 PhD-trained implementation researchers and 30 implementation-experienced stakeholders through SIRC and our international advisory board to participate in a web-based survey. Involving both groups from the outset increases the likely relevance of the measures to stakeholders [2].

##### Data collection and measures

The participants will complete a web-based survey in which the three constructs are defined and the 30 items (10 per construct) are listed in a random order. The participants will assign each item to the construct(s) they perceive the item measures. They will rate their confidence in each assignment from 0 % for “not at all confident” to 100 % for “extremely confident.” Correct assignments (i.e., items assigned to intended constructs) will be coded 1 (a “match”). Incorrect assignments will be coded −1 (“no match”). Each assignment will be multiplied by its confidence rating. Thus, weighted assignments will range from −1 to 1. To assess inter-rater agreement in item allocation *across* constructs, for each expert, we will assign items to the constructs for which the expert gave the highest confidence rating. The resulting data matrices will have 30 rows (i.e., the items) and 30 columns (i.e., the experts). To assess inter-rater agreement in item allocation *within* constructs, we will score the 30 items a 1 or 0 for each construct separately (reflecting whether or not it was assigned to the construct) and for each expert separately. The resulting data matrices will have 30 rows (i.e., the items) and 30 columns (i.e., the experts).

##### Data analysis

To assess substantive validity, we will use one-sample, one-tailed *t* tests to determine whether the item represents the intended construct. The estimated power for a one-sample, one-tailed *t* test with 60 participants and a medium effect size (Cohen’s d = 0.5) is 98.5 %. To formally assess the validity of the implementation outcome conceptual model, we will test a three-factor confirmatory factor analysis model (CFA) wherein each set of 10-item measures will be loaded onto their respective latent factors (i.e., acceptability, appropriateness, or feasibility), with any non-significant correlations or indicator variable factor loadings of <0.50 trimmed from the final model. Power analysis evaluating overall model fit was conducted using the root mean square error of approximation (RMSEA) test of close fit [25], which assumes a null hypothesis of close fit (RMSEA = 0.05) and an alternative hypothesis determination of marginal fit (RMSEA = 0.08) estimates power at 84.6 % with a sample size of 60 participants (sample described above).

#### Structural validity

Structural validity refers to the extent to which the inter-relationships among items measuring a construct in accord with the expected internal structure of the construct [26]. We *predict* three outcomes: (1) the constructs can be measured as unitary factors; (2) the measures are correlated yet distinct; (3) the measures can detect known differences in construct levels, a form of known-groups validity.

##### Design and sample

We will use a 3 × 2 factorial between-subjects design in which we manipulate the three constructs at two levels (high versus low) in vignettes depicting a mental health clinic about to adopt a new evidence-based assessment tool (e.g., Patient Health Questionnaire) and the reactions of stakeholders working there. We will recruit a convenience sample of 240 counselors belonging to the American Mental Health Counselors Association (AMHCA).

##### Data collection and measures

In a web-based survey, the participants will read one of six randomly assigned vignettes and rate the acceptability, appropriateness, and feasibility of using the new assessment tool as they believe the stakeholders at the clinic would rate it. Items will be randomly ordered within and across vignettes to avoid ordering effects.

##### Data analysis

We will test the first two predictions by fitting a three-factor CFA model, forcing items to load on a specified factor but allowing factors to correlate. We will use standardized factor loadings to construct scales and compute correlations among scales, corrected for attenuation due to measurement error. Using the RMSEA test of close fit [25], the estimated power with a sample size of 240 is 100 %. Correlations less than 0.85 will be taken as evidence of discriminant validity. To test the third hypothesis, we will conduct a 3 × 2 analysis of variance (ANOVA) to determine whether the constructs varied as expected by the manipulation of information in the vignettes; the achieved power for this analytic test with 240 participants and a medium effect size (*f* = 0.25) is 85.7 %. Significant main effects will indicate that the participants could distinguish high versus low levels of the three constructs.

#### Reliability assessment

Reliability, the precision of a measure, is a necessary condition for validity [27]. We predict that our measures will demonstrate sufficient inter-item [21] and test-retest reliability. We also predict that our measures will demonstrate sensitivity to change, one of Glasgow and Riley’s required criteria for pragmatic measures [2].

##### Design and sample

We will use a variant of a 3 × 2 factorial within-subjects design in which half of the study participants in the structural validity test will be randomly assigned to receive the same vignette they received earlier (test-retest reliability); the other half will be assigned to receive the exact opposite vignette they received earlier (sensitivity to change). For example, a participant who earlier received a vignette in which the factors were high-high-low would receive a vignette in which the factors are low-low-high. We will stratify the random assignment across vignettes to ensure balance in the conditions for the test-retest and sensitivity analysis.

##### Data collection and measures

We will use the same data collection procedures and measures that we used in the structural validity test, with an additional question about how long it took to complete the ratings (a pragmatic measure should take less than 2 min to complete) [2]. The web-based survey will be sent 5 days following the structural validity test. This interval should minimize practice and fatigue effects.

##### Data analysis

We will assess inter-item consistency using Cronbach’s alpha. Scales demonstrating alpha coefficients greater than 0.70 will be considered reliable. We will assess test-retest reliability using paired *t* tests and Pearson correlation coefficients. Scales demonstrating non-significant paired *t* test and correlations greater than 0.70 will be considered reliable. We will assess sensitivity to change using repeated measures ANOVA with one between-group factor at two levels (same vignette versus opposite vignette) and one within-group factor at two levels (first versus second survey administration).

#### Predictive validity

Predictive validity refers to the extent to which a score on a test predicts a score on a criterion [28]. Proctor and colleagues indicate that acceptability, appropriateness, and feasibility are most salient in the adoption phase of the innovation-decision process [17, 29]. Therefore, we hypothesize that positive perceptions of acceptability, appropriateness, and feasibility are associated with adoption and early use of an innovation or EBP. We will test the predictive validity of our measures in a provider-based research network that routinely adopts multiple innovations and EBPs every year in clinical trials and implements trial results into clinical practice.

##### Study setting

The Community Clinical Oncology Program network (CCOP) is a national provider-based research network that engages community-based physicians in National Cancer Institute (NCI) clinical trials in order to advance the science of cancer prevention, control, and treatment and translate research results into better care [30]. In 2014, the CCOP network combined with the NCI Community Cancer Centers Program to form a new network, the NCI Community Oncology Research Program (NCORP). NCORP physicians choose which clinical trials to adopt and use (i.e., offer to their patients and enroll them).

##### Design and sample

We will use a cross-sectional design and sampling frame of 2520 NCORP physicians in three oncology specialties: hematology, medical, and radiation. Using a list of NCORP physicians provided by Nation Cancer Institute Division of Cancer Prevention (NCI/DCP) and data from the American Medical Association (AMA) Physician Masterfile (see below), we will draw a random sample of 600 active NCORP physicians, stratified by NCORP organization and medical specialty.

##### Data sources and measures

We will survey physicians to obtain data on their perceptions of acceptability, appropriateness, and feasibility of an innovative clinical practice embedded in a clinical trial. We will obtain data on the physicians’ adoption and use of the clinical trial from the NCORP organizations’ administrators, who routinely report such data to NCI/DCP. We will measure *adoption* as a binary variable indicating whether a physician enrolled any patients on the clinical trial and use as the number of patients a physician enrolled. We will obtain data on physicians’ demographics, medical specialty, and years in practice from the AMA Physician Masterfile, which includes data for more than 1.4 million physicians, residents, and medical students in the US.

##### Data collection

We will identify for each oncology specialty a clinical trial that departs significantly from routine clinical practice (i.e., an innovation). Shortly after NCORP organizations activate the trials, we will mail a survey to the sampled physicians asking them to rate the acceptability, appropriateness, and feasibility of enrolling their patients in the trial.

##### Data analysis

We will conduct a three-factor CFA, create scales from standardized factor loadings, compute inter-item consistency for the scales, and compute correlations among scales corrected for attenuation due to measurement error. These results will indicate whether our measures exhibit comparable structural validity, reliability, and discriminant validity in the field as they do in the lab. We will test our hypothesis by testing a structural equation model in M*plus* 7 [31] using the three latent factors from the three-factor CFA as exogenous predictors, and adoption and use as the endogenous outcomes. The estimated power [25] for the model is 100 %.

### Aim 3: identify CFIR- and IOF-linked measures that demonstrate both psychometric and pragmatic strength

#### Introduction

The objective of Aim 3 is to establish the psychometric and pragmatic quality of quantitative measures used in implementation science. We predict that only measures of implementation outcomes [17], and not those of other implementation-relevant constructs, will be both high in psychometric quality and pragmatic strength (Fig. 1). To test our prediction, quantitative measures of CFIR [16] and IOF [17] constructs will be rated to establish psychometric and pragmatic strength.

#### Research design

Building on our preliminary work, we will (a) refine the EBA criteria, (b) extract the relevant data from the literature, (c) rate each measure using the EBA criteria, and (d) summarize the data.

#### Refine evidence-based assessment criteria

We will modify our established EBA rating criteria in two ways. First, we will add specifiers to the predictive validity criterion to clarify which implementation outcome (*acceptability*, *feasibility*, *appropriateness*, *adoption*, *penetration*, *cost*, *sustainment*, *fidelity*) measures have been demonstrated to predict. Second, we will add the pragmatic rating criteria from Aim 1 to reveal critical gaps in the implementation measures landscape.

#### Data extraction (cleaning, coding): highlighting EBA relevant data

Our data extraction protocol is based on the EBA criteria in that trained research assistants will use a software program to highlight and comment on information pertaining to psychometric and pragmatic properties in original research reports that directly align with each of the EBA criterion.

#### Data analysis: rating data obtained through extraction

The trained research specialists and task force members will rate the psychometric and pragmatic quality of measures by applying the refined EBA criteria to the highlighted measure packets and submitting their ratings via an online survey. The research specialists will rate all measures. The task force members will only rate measures with moderate evidence of reliability (e.g., internal consistency) and validity (e.g., structural validity) as determined by the research specialists. Based on our pilot work, we anticipate that no more than 200 measures will meet this moderate evidence of psychometric quality. We will adopt a “worst score counts” methodology [32] when applying the EBA criteria. This implies that when rating a measure based on multiple original research articles, the worst score obtained on internal consistency, for instance, would be the “final” rating the measure would receive on that criterion. Two-point discrepancies between research specialist and task force member ratings will be resolved by the investigative team.

#### Data summarization and interpretation

The EBA criteria have quantitative anchors associated with the qualitative descriptions (e.g., “no evidence” = 0, “minimal/emerging” = 1, “good” = 2). This allows for the generation of head-to-head comparisons of measures within constructs, which will serve as a tool for researchers and stakeholders in identifying psychometrically strong, pragmatic measures. To inform the measurement-focused research agenda (which constructs and domains are saturated with high-quality, pragmatic measures; which are underdeveloped), we will summarize the EBA ratings across measures within domains and constructs and locate each construct within the four quadrants (see Fig. 1). We will interpret the measure quality profiles and the results of the four-quadrant grid in collaboration with our international and stakeholder advisory boards.

### Trial status

At the time of submission, both the international advisory board and the implementation scientist task force have been engaged. The stakeholder panel is formed and nominations for our stakeholder task force have been collected and rank ordered to achieve balance in our representativeness.

## Discussion

### Innovation

No research, to our knowledge, has attempted to systematically produce and identify pragmatic implementation science measures. In addition, the only existing pragmatic criteria were generated in the absence of stakeholder involvement. Pragmatic implementation science measures could be useful for assessing organizational needs and contexts (to select and deploy implementation strategies), monitoring strategy impacts, and refining implementation processes to improve outcomes. The resulting rating criteria are innovative because they will provide a method for systematically evaluating pragmatic measure strength, which will inform measure development, evaluation, and selection.

In addition, the aim to develop new measures of implementation outcomes, focusing on acceptability, appropriateness, and feasibility is innovative because no valid, reliable, pragmatic measures exist for these constructs. Given their implications for predicting the likelihood of uptake of EBPs, these constructs are critically in need of systematic measurement development. This work will also establish a novel, rigorous method for measure development that takes into account pragmatic measure qualities.

Finally, the proposed evaluation of a comprehensive array of existing measures is innovative because it moves beyond the traditional approach of evaluating measures based only on their psychometric properties and will reveal four quadrants (Fig. 1) delineating measure strength. Although other reviews of measurement have been published [4, 5], no systematic review, empirical rating of this scale and degree of comprehensiveness has been conducted in implementation science.

### Limitations

There are several limitations to the current studies. First, the pragmatic construct definition may carry focused relevance for mental and behavioral health settings, given the settings represented by participating stakeholders. Given our preliminary literature review, it is anticipated that pragmatic measures qualities are relatively consistent across settings; however, this is an empirical question. Second, it is possible that being able to clearly delineate pragmatic dimensions and sub-dimensions may prove difficult; however, by using both Q-sort and Delphi methodologies we will be able to impose structure across the data. In addition, the three new implementation outcome measures may be less of a fit for health-focused settings; however, by assessing predictive validity among oncologists, we will be increasing the likelihood of measure relevance. Finally, the review of measures is limited to the CFIR and IOF-linked constructs. These frameworks were selected given their clear impact on the field (with respect to citation rate) and their relatively comprehensive coverage. Having said that, numerous frameworks have emerged in recent years reflecting unique constructs for which independent systematic measure reviews are likely necessary.

### Impact

Identification and application of evidence-based implementation strategies requires the availability of psychometrically strong, pragmatic measures. The interrelated set of aims in the current project will yield methods and measures for advancing the field. The resulting pragmatic rating criteria may gain widespread use, not only in implementation science but also in related fields. For example, Beidas et al. [33] recently used Glasgow and Riley’s pragmatic criteria [2] as inclusion criteria for a review of patient symptom measures. In addition, the development of three new reliable, valid, and pragmatic measures of implementation outcomes will position researchers to identify strategies that promote adoption. The comprehensive assessment of psychometric and pragmatic qualities of over 420 measures will enable researchers and stakeholders to make use of high-quality existing measures and contribute new measures in underdeveloped areas. Moreover, our work may reveal measurement-focused reporting standards. This proposal reflects necessary steps in our research program with the eventual goal of developing an online battery of reliable, valid, and pragmatic implementation science measures. With SIRC as our platform (>520 international members) and the close involvement of nine world-renowned implementation scientists, the findings and outcomes of this set of studies have the potential for high impact.

## Abbreviations

AMA: American Medical Association
AMHCA: American Mental Health Counselors Association
ANOVA: analysis of variance
CCOP: Community Clinical Oncology Program
CFA: confirmatory factor analysis
CFI: comparative fit index
CFIR: Consolidated Framework for Implementation Research
EBA: evidence-based assessment
EBP: evidence-based practice
GEM: Grid-Enabled Measures
ICC: intraclass correlation
IOF: Implementation Outcomes Framework
NCI: National Cancer Institute
NCI/DCP: Nation Cancer Institute Division of Cancer Prevention
NIH: National Institute of Health
NIMH: National Institute of Mental Health
RMSEA: root mean square error of approximation
SIRC: Society for Implementation Research Collaboration
SRMR: standard root mean square residual
